# Spatiotemporal Patterns of African Swine Fever in Wild Boar in the Russian Federation (2007–2022): Using Clustering Tools for Revealing High-Risk Areas

**DOI:** 10.3390/ani13193081

**Published:** 2023-10-02

**Authors:** Olga I. Zakharova, Fedor I. Korennoy, Ivan V. Yashin, Olga A. Burova, Elena A. Liskova, Nadezhda A. Gladkova, Irina V. Razheva, Andrey A. Blokhin

**Affiliations:** 1 Federal Research Center for Virology and Microbiology, Branch in Nizhny Novgorod, Nizhny Novgorod 603950, Russia; korennoy@arriah.ru (F.I.K.); ivanyashin@yandex.ru (I.V.Y.); burovaolga@list.ru (O.A.B.); liskovaea@mail.ru (E.A.L.); nivigladkova@yandex.ru (N.A.G.); razheva64@bk.ru (I.V.R.); and.bloxin2010@yandex.ru (A.A.B.); 2 Federal Center for Animal Health (FGBI ARRIAH), Vladimir 600901, Russia

**Keywords:** wild boar, animal population density, African swine fever, hot spot analysis, spatiotemporal clustering, SaTScan, Russia

## Abstract

**Simple Summary:**

In this study, we utilized the methods of spatiotemporal analysis to reveal and visualize the areas in the Russian Federation where an increased density of wild boar population might be related to the concentration of African swine fever (ASF) cases in wild boar. We demonstrated the areas (at the municipality level), where the elevated wild boar population density has continued to rise in recent years, that may be treated as high-risk areas, subject to the application of enhanced surveillance and population control measures.

**Abstract:**

African swine fever (ASF) is an infectious disease that affects both domestic pigs (DPs) and wild boar (WB). The WB population plays an important role in the spread of ASF as the WB acts as a natural reservoir of the virus and transmits it to other susceptible wild and domestic pigs. Our study was aimed at revealing the areas with a high concentration of the WB population, and their potential relationships with the grouping of ASF cases in WB during the course of the ASF spread in the Russian Federation (2007–2022). We collected the annual data on WB numbers by municipalities within the regions of the most intensive ASF spread. We then conducted spatiotemporal analysis to identify clustering areas of ASF cases and compare them with the territories with a high density of WB population. We found that some of the territories with elevated ASF incidence in WB demonstrated spatial and temporal coincidence with the areas with a high WB population density. We also visualized the zones (“emerging hot spots”) with a statistically significant rise in the WB population density in recent years, which may be treated as areas of paramount importance for the application of surveillance measures and WB population control.

## 1. Introduction

African swine fever (ASF) is a contagious viral disease in domestic and wild pigs [1,2,3]. It causes high fever, loss of appetite, weakness, and ultimately, death in infected animals. The ASF virus can survive for long periods of time in both pork products and the environment, making it difficult to eradicate the disease [4,5,6]. There is currently no vaccine or cure for African swine fever, and the main method of prevention is through strict biosecurity measures and monitoring of wild boar and livestock pig populations [7].

ASF is an ongoing problem for many countries due to the transboundary spread of the disease and the enormous economic damage that arises from the costs associated with restrictions on the trade and movement of pigs and pork products, as well as the hunting ban and the implementation of veterinary and other quarantine measures in the outbreak areas [5].

The circulation of infection within the population of wild boar is a part of the African swine fever epidemic, promoting the formation of natural foci of the disease and its further spread to unaffected areas [8,9]. The presence of wild boar in the ecosystem is of great importance in the transmission of the African swine fever virus both among wild boar individuals and to populations of domestic pigs, which is recognized by many studies [10,11,12].

For the regions of the Russian Federation, the circulation of African swine fever in the wild boar population is typical. Recently, such a mechanism of an epidemic disease has been clearly traced in the Far East [13,14].

In addition to the density of the wild boar population, factors such as the stability of the ASF virus in wild boar carcasses, the social structure of the population, geography, and many others may also affect the transmission process of the virus [15,16,17]. The wild boar is a social animal and can therefore group together even in low-density regions, resulting in geographical areas with a higher local concentration of animals and creating conditions for the emergence of new African swine fever epidemics [7,18].

The emergence of ASF outbreaks in wild boar from 2007 to 2022 has been mainly recorded sporadically as isolated occurrences, rarely as sweeping short-duration epidemics. Ecological factors that contribute to the preservation of the ASF virus in the environment make it difficult to eradicate the disease in the wild boar population [19,20,21]. Monitoring the wild boar population is an important component of surveillance and control when planning preventive measures [22]. The monitoring activity is used to timely detect disease cases and to apply effective measures to prevent the spread of infection to unaffected areas [23,24]. The density of the wild boar population varies dynamically as a result of active depopulation measures as well as boars’ migration. Thus, accounting for these changes as well as their visualization provide important information for decision-making in the field of animal disease control [25,26,27]. It is necessary to accurately estimate the population density of wild boars in specific areas in order to determine the volume of removal of this species [28]. Modern information technologies, particularly geo-information systems (GIS) available in various areas of science, allow for the automatic data processing, display, and analysis of information in a spatial-temporal format.

The analysis of spatial-temporal patterns of disease occurrence in relation to the susceptible population density allows for determining and evaluating areas (or foci) where the active transmission of pathogens occurs, which potentially may contribute to the assessment of introduction and spread risks [29,30,31]. In the Russian Federation, the methods of planned monitoring, accounting, and assessment of wild boar populations traditionally depend on the geographic location of protected areas and hunting grounds and represent a government system of observations and evaluations [32]. Due to the current epidemic situation of African swine fever in Russia, comprehensive studies of the spatial-temporal structure and dynamics of the wild boar population density at local and regional scales are especially relevant today. The counting, analysis, and visualization of the actual size of susceptible animal populations are important for making informed management decisions aimed at regulating the population density and to determine strategies for disease prevention and elimination.

Therefore, our study aimed to investigate and analyze the spatial-temporal patterns of African swine fever (ASF) cases depending on the density of the susceptible wild boar population by municipalities of affected regions in Russia, and to reveal and visualize hot spots of increased wild boar population density through 2007–2022, which may indicate the areas for the paramount application of monitoring and risk-reduction efforts.

## 2. Materials and Methods

### 2.1. Study Area

**Figure 1:**
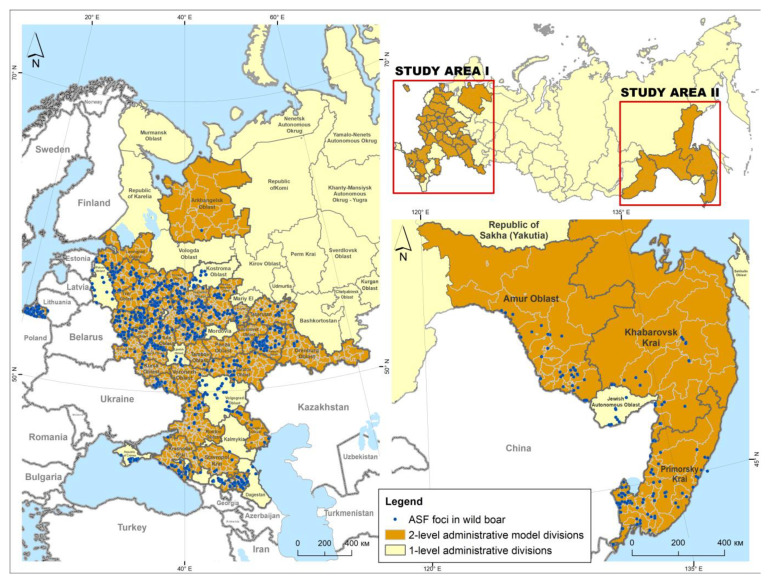
The African swine fever foci in wild boar population in the model regions of the Russian Federation from 2007 to 2022.

The study area was determined by the regions of the Russian Federation where (1) ASF outbreaks in wild boar were recorded within the study period of 2007–2022, and (2) annual data on wild boar population were available at the municipality level (Figure 1). The entire model region consisted of 39 subjects (first-level administrative divisions). It was divided into two zones, namely European (35 subjects) and Far Eastern (4 subjects). The model subjects consisted of 2440 s-level divisions (municipalities or districts).

### 2.2. Data Sources

Data on African Swine Fever outbreaks in the Russian Federation were obtained from the WOAH World Animal Health Information System (WAHIS) for the period from 2007 to 2022 [33]. In total, 608 foci of African swine fever in the population of wild boar in the European zone and 103 foci in the Far Eastern region were included in the analysis. In this study, we considered an “outbreak” as a notified occurrence of dead or infected wild boar(s) with laboratory-confirmed ASF, determined by geographic coordinates and the date of occurrence. A single dead or infected wild boar was referred to as a “case”.

Data on the wild boar population size in the studied municipalities of the model regions annually from 2007 to 2022 were obtained through requests from the regional administrations of the Ministries of Natural Resources and Ecology of the Russian Federation [32]. The data on the wild boar population size are estimated indicators that have been extrapolated to the entire territory of the district. One of the main methods used for monitoring the population of wild boars in the regions of the model territories is winter route counting, which is currently applied across Russia wherever there is stable snow cover. This method provides a general characteristic of the population in the area and is widely used in hunting farms throughout Russia.

### 2.3. Spatiotemporal Cluster Analysis of ASF Cases in the Wild Boar

The study included three stages:

(a) Identifying spatial-temporal clusters of African swine fever incidence in wild boars in the model regions from 2007 to 2022, depending on animal density using a discrete Poisson model;
(b) Identifying spatial-temporal clusters of increased wild boar population density in the model regions from 2007 to 2022 using a normal probability model;
(c) Identifying “hot and cold spots”, i.e., geographic areas with increasing and decreasing trends in wild boar population density, using the Emerging Hot Spot Analysis (EHSA) method.

The search for spatial-temporal clusters was conducted using the Kulldorff spatial-temporal scan statistic [34,35]. The method scans the whole study area with a cylindrical scanning window of changing radius and height, where the height represents time. The number of cases (or associated attributes) within each candidate cluster are compared with the one expected under a null hypothesis. The maximum spatial and temporal search size for clusters were taken by default for 50% of the study area and time period, respectively. Windows with a significant excess of observed cases compared to expected ones are reported as spatiotemporal clusters. Municipalities of the 2nd order (districts) were used as spatial modeling units. The annual number of ASF cases in each district and the population density of wild boars were linked to the centroid of the corresponding district. Centroids were obtained using the Feature to Point tool in ArcGIS Pro (Version 2.9).

#### 2.3.1. ASF Incidence Cluster Analysis with Poisson Model

A discrete spatial-temporal Poisson probability model was used to identify clusters of increased ASF incidence in the wild boar population. The null hypothesis for this model assumes that the expected number of disease cases within some areas is Poisson distributed depending on the population size (i.e., is proportional to the wild boar population size) at the same area [34,36,37]. The *p*-value for all clusters was estimated using 999 Monte Carlo simulations. Clusters with *p* < 0.05 were considered significant [38].

#### 2.3.2. Wild Boar Density Cluster Analysis with Normal Probability Model

To identify distribution patterns of areas with high wild boar density, a spatial-temporal cluster analysis was performed using a normal probability model [39]. This model is used to analyze continuously distributed data, assuming that the theoretical (expected) distribution is normal. The model identifies clusters with a statistically significant (*p* < 0.05) excess of population density over the expected value [40].

### 2.4. Emerging Hot Spot Analysis (EHSA) of Annual Wild Boar Population Density

Since the results obtained from SaTScan software may be relatively unstable, being strongly dependent on the selected size of the scanning window [41], hot and cold spot analysis was also conducted to justify the areas with certain groupings of high/low animal density and trends in its temporal changes [42].

To perform EHSA, the data on the wild boar population density were represented as a spatiotemporal structure referred to as a “cube,” where the spatial units were the analyzed municipalities with wild boar density data for the sufficient number of years (*n* = 1187 in the European zone and *n* = 82 in the Far Eastern zone), and the vertical dimension represented time with a yearly interval.

The primary objects of analysis are time-series data, known as “population density bins,” for each municipality over the entire study period. The EHSA method combines two statistical indicators: the Getis–Ord Gi* hot spot statistic test, which determines the location and intensity of spatial clusters of wild boar density (hot and cold spots), and the Mann–Kendall statistic to evaluate the temporal trends in hot and cold spots’ emergence. The statistical significance of hot and cold spots, as well as their trends, is determined based on the variance of the time-series values [43,44,45].

The results of the hot and cold spot analysis were visualized based on z-scores and corresponding *p*-value significance criteria. Seventeen categories of hot and cold spots were identified depending on the temporal trends in their formation (Table S1): new, consecutive, intensifying, constant, decreasing, sporadic, fluctuating, and historical. For example, a “new” hot spot indicates the formation of a high-density wild boar cluster in recent years of the study period. A “constant” hot spot indicates a consistently observed high wild boar density in the area throughout the study years.

We compared clusters of increased wild boar population density with clusters of increased ASF incidence, based on the principle of spatial and temporal intersection. Additionally, the identified hot and cold spots were used to reveal the geographic territories with increasing and decreasing trends in the wild boar population density and areas with intensive hunting resource utilization.

### 2.5. Software

Spatiotemporal cluster analysis was conducted using SaTScan software version 9.7 [40]. Statistical data processing was performed using the MS Office Excel v.1 application package. Emerging Hot and Cold Spot Analysis (EHSA), spatial data processing, and mapping of results were carried out using ArcGIS Pro (Esri, Redlands, CA, USA).

## 3. Results

### 3.1. Descriptive Analysis

**Figure 2:**
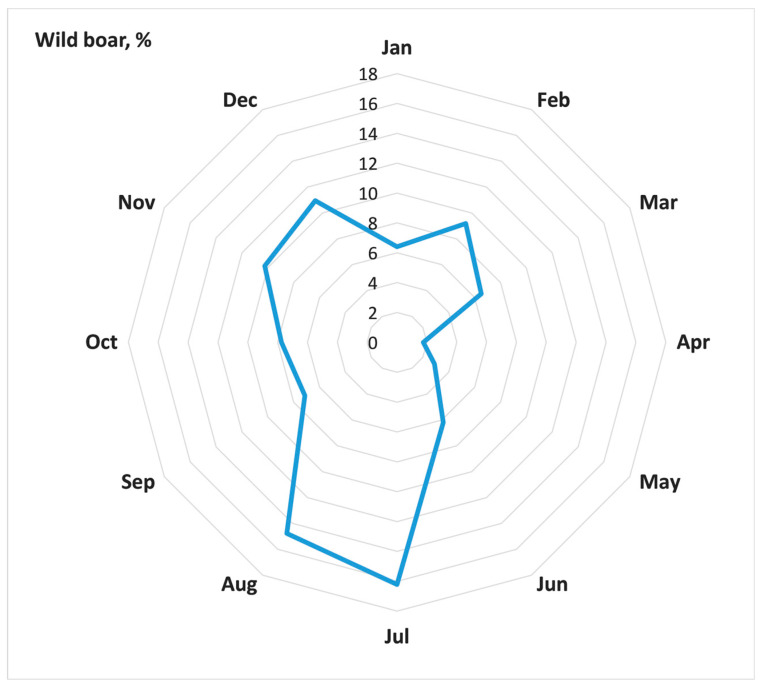
Seasonality of ASF outbreaks in wild boar within model district of Russia, 2007–2022 (a share of outbreaks in a particular month in the total number of outbreaks).

The number of ASF outbreaks among the population of wild boars from 2007 to 2022 accounted for 42.5% of all ASF outbreaks in Russia during this period (2678). ASF outbreaks in the wild boar population demonstrate a pronounced seasonality, when peaks occur in November and December during the cold season and July and August during the summer season (Figure 2).

### 3.2. Spatiotemporal Cluster Analysis of ASF Foci in Wild Boar Population Density

**Figure 3:**
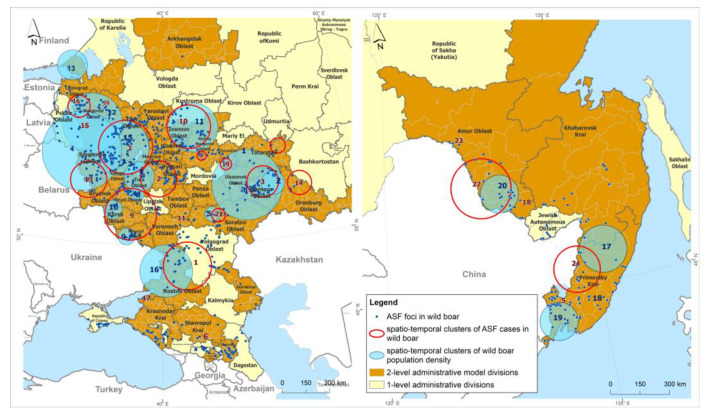
Spatiotemporal clusters of ASF cases in wild boar, and spatiotemporal clusters of districts with high wild boar population density in the Russian Federation, 2007–2022.

Analysis of spatial-temporal clusters of ASF incidence in populations of wild boar in the model regions of the Russian Federation from 2007 to 2022 using a Poisson probability model revealed 24 statistically significant clusters with significantly different durations of formation ranging from 1 year to 9 years (Figure 3, Table 1).

In total, 2 prolonged clusters (clusters #10 and #16), with a duration of more than 7 years, 10 short-term clusters, with durations of up to 1 year, and 12 intermediate clusters, with a duration of 2 to 6 years, were identified.

The cluster of African swine fever foci #16, which coincided in the period from 2011 to 2016 with density cluster #12, was prolonged (duration time amounted to 9 years), although its relative risk was not particularly high and amounted to 199.

Clusters #4, 5, and 6 had moderate geographic areas within the radius of one district and were short-lived (duration time from 1 to 3 years) but presented the highest risk of infection spread due to the high density of susceptible animal populations (ODE4 = 1523.59, ODE5 = 2013.61, ODE6 = 1947.59).

Areas of high risk for registered outbreaks of ASF can also be identified in clusters #11 and #17, where the ODE was 1915.12 and 286.29, respectively.

As a result of cluster analysis of the yearly wild boar population density data, 20 clusters were identified, including 4 significant clusters that combine areas of the Russian Far East (Figure 3, Table 2). Cluster number 19, with the highest LLR of 181.86, is located in Primorsky Krai and consists of 13 districts where the wild boar density remains high at 5.27 head/km^2^. The wild boar density in the areas within cluster number 17, located in the territories of Primorsky and Khabarovsk Krai, was lower at 4.27 head/km^2^.

In the central zone of the Russian Federation, statistically significant clusters, numbers 3 and 7, with LLR values of 169.98 and 199.74, respectively, are locally located in two adjacent districts, Medynsky and Dzerzhinsky, in the Kaluga region, where the wild boar density had the highest values of 1.89 head/km^2^ and 2.02 head/km^2^, respectively (Table 2).

Cluster numbers 1, 5, and 9, located in the territories of 15 districts (Republic of Tatarstan, Saratov, and Belgorod regions), had wild boar densities of 0.88, 0.47, and 0.35 head/km^2^, respectively, exceeding the threshold wild boar density of 0.025 head/km^2^ recommended by the ASF elimination plan [46] by 353%, 189%, and 140%, respectively.

The results of the spatial-temporal analysis using the normal probability model are presented in Table 2.

The #5 cluster for ASF cases, located in Primorsky Krai, had a temporal coincidence with cluster #19 in the animal population density, which demonstrated a high density of individuals in the areas forming the cluster—5.27 head/km^2^. This could be a potential risk factor for the concentration of ASF outbreaks in the region in 2020.

Cluster #10 for ASF cases among wild boars, encompassing territories in the Nizhny Novgorod, Vladimir, Ivanovo, and Kostroma regions, coincided in the time of formation with cluster #11 for population density (2016), identified by the normal probability method. The density of wild boars in that cluster was 0.21 head/km^2^.

Cluster #3 for ASF cases, located in the Samara region and with a temporal period in 2020, was the shortest major epidemic, resulting in over 60 ASF foci registered among wild boars and coincided with cluster #6 for population density, in which the animal density was 1.88 head/km^2^.

### 3.3. Emerging Hot Spot Analysis of Wild Boar Population Density

**Figure 4:**
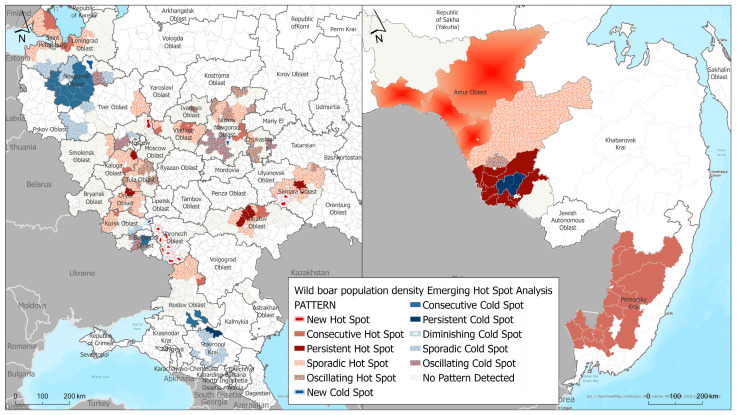
Results of Emerging Hot Spot Analysis of wild boar population density in European part (**left**) and Far East (**right**) of the Russian Federation.

As a result of hot and cold spot analysis, typical areas were identified in the central European zone of the Russian Federation and in the southeastern part of the country with an increased/decreased concentration in the wild boar population density, and classified as sporadic, persistent, new, and fluctuating depending on the temporal patterns of clustering (Figure 4). Sporadic and persistent hot spots in the wild boar population concentration were identified in the northwest part of Russia, in the Leningrad region bordering Finland and the Baltic States. Territories in the central part of the Russian Federation, specifically in Vladimir, Tver, Nizhny Novgorod regions, the Republic of Chuvashia, and the western and southwestern parts such as Smolensk, Kaluga, Tula, Orel, and Kursk regions, are characterized by temporary increases in the animal population density and likely by the persistence of the African swine fever (ASF) virus in the environment fomites, including in the carcasses and remains of infected wild boars.

We also identified cold spot areas, where changes toward a decrease in the population density were observed during the analyzed period or no dynamics were observed. Cold spots were identified in the southern part of the Russian Federation—in the Stavropol Krai, Rostov, Tver, Novgorod, and southern parts of the Leningrad Oblasts. These areas likely experience constant or periodic decreases in the wild boar density population.

In the Far East part of the Russian Federation, where mainly hot spots were revealed, a persistent cold spot was identified, presenting an area where the population density remained at the same level or constantly decreased as a result of measures being taken to regulate the wild boar population. This cold spot is located in the eastern part of the Amur Oblast, in the Romnensky and Zavitinsky districts. Characteristics of the obtained hot and cold spots (geographic locations) are presented in the Supplementary Material (Table S1).

**Figure 5:**
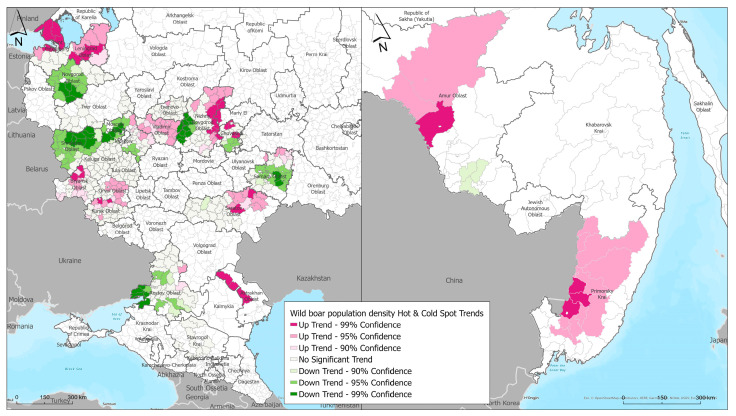
Temporal trends in wild boar population density hot spots across in European part (**left**) and Far East (**right**) of the Russian Federation.

Territorial trends in the increasing wild boar population density were identified in the central European zone of the Russian Federation in subjects such as Vladimir, Moscow, Kaluga, Bryansk, Oryol, Kursk, and Nizhny Novgorod regions, the Mari El Republic, as well as in the southern and southeastern parts of Russia in the Rostov, Samara, Saratov, and Astrakhan regions. A trend in the increasing wild boar population density was also noted in the Leningrad region in the northwest of Russia (Figure 5).

In the Far Eastern region of Russia, a trend in the increasing wild boar population density was noted in Amur Oblast and Primorsky Krai.

Territories with a decreasing trend in the wild boar population density have been identified in the central part of the European part of the Russian Federation in the Novgorod and Smolensk regions, the western part of the Nizhny Novgorod, Moscow, and Rostov regions, and the central part of the Samara region. Such a change in the population density is likely linked to measures being taken to regulate the number of wild boar.

## 4. Discussion

Animal infectious diseases cause enormous damage to the food security of many countries, resulting in significant financial losses due to the mass culling of animals [47,48]. As a reliable vaccine against ASF has not yet been developed, the main methods of combating ASF involve implementing strict biosecurity measures in the management of pigs and wild boar [3,7,49]. This includes compartmentalization on pig farms based on zoosanitary status, and preventive measures aimed at regulating the population density of susceptible animals [50].

There is no standard strategy for controlling ASF epidemics that could be applied to all ASF affected areas due to the different geographic and regional characteristics of populations of wild boars [10,51,52]. Measures to prevent the introduction of the ASF virus from an affected region to unaffected region are a preferable option for standard prevention measures [53,54].

The spread of ASF in the wild boar population in the affected districts in the Russian Federation has distinct characteristics observed in the dynamics of epidemics [55,56]. The incidence of ASF among the wild boar population exhibits a seasonality of outbreak registrations [57]. The new foci of ASF were recorded during both winter peaks in November and December, and summer peaks in July and August. It is possible that the winter peak in ASF incidence is due to the biological characteristics of the animals, such as the mating season and herd re-groupings. The summer peak is likely caused by human activities, such as frequent visits to forests and hunting activities. An indirect contact between contaminated pork products or fomites left in the field can be a source of infection in the wild boar population in proximity to domestic pig farms, which presumably may happen mainly in the periods of high economic activity in the population, i.e., in the summer season.

Spatial and spatiotemporal methods for identifying areas of concentration (clusters) of disease outbreaks have an important role in modern epidemiological research, as well as in the practice of public health and preventive veterinary medicine. Their use enables the identification of potential etiological and pathogenic causes of epidemics and determines the key ways to address the disease eradication.

Therefore, in our study, we aimed to demonstrate the necessity of using cluster analysis methods to identify areas with a concentration of ASF foci in wild boars depending on the localization of areas with a high density of populations. We conducted a comparative analysis of geographic regions forming these clusters based on the animal population density and identified high-risk areas where the density of the wild boar population remains elevated.

Spatial-temporal analysis of ASF foci in the wild boar population using a Poisson probability model that took into account the density of the wild boar population revealed clusters with a high risk of detecting new foci of the disease in the central part of the European Zone of the Russian Federation.

The ASF incidence clusters in the wild boar population had different temporal durations, with some epidemics characterized by the formation of short-term clusters with a time duration up to 1 year, as well as prolonged clusters lasting up to 9 years. The trend toward the formation of short-duration clusters with a high risk of new foci is likely due to the prolonged epidemic situation among wild boars and the expansion of ASF disease to previously unaffected territories of the Russian Federation.

Geographically, ASF hot spots characterized by high relative risk values were localized in subjects in the central part of European Russia. The spatial-temporal spread of ASF was characterized by the movement of cluster territories from Cluster #6 in 2010 toward the north to Cluster #17 in 2012, and then to Clusters #11 (2021) and #4 (2015). In 2022, a high-risk cluster #5 was identified in Primorsky Krai in the Far East region, which may be associated with the prohibition of wild boar depopulation in the bordering territories of China with Russia.

The distinguishing epidemiological feature of the identified clusters was the duration of their existence, with some having a short period of 1 or 2 years, while others were long-lasting for 7 years or more (clusters #6 and #17). This can be explained by the fact that outbreaks of African swine fever (ASF) in wild boar populations in different regions of Russia have had varying durations, ranging from widespread epidemics in a short period, such as in the Samara region in 2020, where more than 60 foci of ASF in carcasses of infected wild boars were registered [31], to sporadic outbreaks that are predominantly found in the most affected regions of the Russian Federation [58].

Therefore, there is a view among international scientists that even at a very low population density of wild boars, there is a window of uncertainty when African swine fever (ASF) is still present among animals, but practically undetected by standard diagnostic methods, which complicates any further disease management, including possible infection-elimination strategies [4,7,10].

As a result of a spatial-temporal analysis of geographic areas where foci of African swine fever (ASF) were registered from 2007 to 2022, with a predisposing factor likely being the increased population density of wild boar, several clusters were identified in the northwestern, central, and southeastern regions of the Russian Federation. The territorial and temporal coincidence of these high-density clusters may be explained by the continued concentration of animals in the ASF foci areas, despite the ongoing disease control measures implemented in the affected regions of Russia, which involve the annual population reduction in wild boars [46].

Based on the estimated density of wild boar populations and through the analysis of emerging hotspots (EHSA), the geographic scope of regions affected by African swine fever (ASF) in the Russian Federation with high densities of wild boars has been determined. These areas have a tendency to experience increases or decreases in animal population density, making them important for conducting targeted measures aimed at regulating boar populations.

As a result of the EHSA analysis, an increasing spatiotemporal trend was observed, which can be attributed to the existing high density of animal populations in the territories of the northwestern and southeastern parts of Russia (with an average density of over 0.85 boars per square kilometer within the trend subjects). The density of wild boars in the subjects of the European zone has been found to be 0.025 animals per square kilometer of hunting grounds, based on the plan for the eradication of African swine fever in the subjects of the Russian Federation.

Areas of risk can be considered territories with a tendency toward the clustering of areas of concentration of animals where this trend is maintained and expressed as an increasing trend in the population density of wild boar. An increasing trend in the population density of wild boar has been observed in the following subjects of the European part of Russia: Leningrad, Vladimir, Kaluga, Bryansk, Oryol, Kursk regions, the north part of the Nizhny Novgorod region, Chuvash Republic, the center of the Saratov region, the northeastern part of the Samara and Moscow regions. In the Far East, areas with high densities of wild boar, such as the Amur region and Primorsky Krai, provide an opportunity to assess areas at risk of ASF occurrence and to some extent represent an example of the transboundary spread of ASF among the population of wild boar.

Currently, depopulation is carried out annually in all problematic regions of Russia, which has an irrational basis due to the high cost of implementing the measures. We have studied some clustering methods for identifying areas with a certain risk of ASF epidemics to more effectively plan measures to regulate the population density of wild boar in the subjects of the Russian Federation.

It should be noted that the data on wild boar population size and density used in this study can be affected by a bias related to imperfect counting methods. Normally, wild boar counts in Russia are obtained via “winter route counting”. However, when this method is used in individual hunting farms and nature reserves (i.e., smaller areas), it often underestimates the population size of wild boar. This is because the counting is conducted in the second half of winter when the movement of wild boar on the ground is disrupted by heavy snow, and their transitional activity is drastically reduced. The obtained assessment data are often extrapolated to the remaining area of the district, which can lead to serious errors.

## 5. Conclusions

The lack of an effective vaccine against ASF currently determines the key points in the strategy for combating and eliminating the infection, which remains a global problem for many countries in the world. Due to the ability of the ASF virus to maintain its virulent properties in the environment for a long period, problem areas are formed in the subjects of the Russian Federation, with the potential for the further spread of the infection. The situation is aggravated by the presence of local areas with a high density of wild boar, which can be risk zones for the spread of ASF. The location and duration of clusters of ASF outbreaks among wild boar in the problematic regions of Russia provide an idea of areas with the most extended epidemics and the presence of a high potential for further persistence of the ASF virus in the wild.

The clustering methods used in this study, which investigate hot spots—areas of high wild boar population density—can be used to determine high-risk territories for the purpose of enhancing active and passive monitoring in certain geographical areas and applying biosecurity measures during hunting, as well as other risk-reduction measures. Knowledge of the identified trends and patterns of ASF spread in the wild boar population enables the improvement of implemented measures within the territorial boundaries of local epidemics. The use of spatial-temporal analysis methods of ASF foci and wild boar population density using clustering tools presents a risk-oriented approach for identifying priority zones (risk territories) and assessing the effectiveness of prevention measures.

## Figures and Tables

**Table 1:** Characteristics of spatiotemporal clusters of ASF incidence in the affected model districts of Russia, 2007–2022.

Cluster #	Start Date	End Date	Cluster Duration (Years)	Relative Risk	ODE	Observed Cases	Expected Cases
1	2010	2014	5	85	68.26	543	7.96
2	2013	2016	4	42	37.05	316	8.53
3	2020	2022	3	92	86.81	148	1.70
4	2015	2015	1	1566	1523.59	74	0.05
5	2020	2022	3	2063	2013.61	65	0.03
6	2010	2010	1	1990	1947.59	58	0.03
7	2012	2016	5	9	7.93	259	32.64
8	2019	2020	2	180	176.84	48	0.27
9	2015	2019	5	9	8.57	127	14.82
10	2016	2022	7	23	23.03	44	1.91
11	2021	2021	1	1924	1915.12	12	0.01
12	2020	2020	1	80	79.55	20	0.25
13	2014	2014	1	9	9.09	48	5.28
14	2020	2020	1	23	22.55	23	1.02
15	2014	2016	3	36	35.53	18	0.51
16	2011	2019	9	199	9.03	32	3.55
17	2012	2012	1	287	286.29	9	0.03
18	2019	2020	2	7	7.15	37	5.17
19	2016	2018	3	35	34.73	15	0.43
20	2012	2012	1	23	23.33	17	0.73
21	2016	2016	1	8	8.32	18	2.16
22	2021	2021	1	5	4.67	29	6.21
23	2020	2021	2	7	6.87	13	1.89
24	2019	2022	4	3	2.63	36	13.70

Note: ODE—observed/expected (the ratio of the observed number of ASF cases to the expected number within a cluster, provided the distribution corresponds to the null hypothesis). Relative Risk (RR)—the relative risk value shows the ratio of observed ASF cases within the cluster to the expected number of cases outside the cluster.

**Table 2:** Characteristics of spatiotemporal clusters of wild boar population density in the model districts of Russia, 2010–2022.

Cluster #	Start Date	End Date	Cluster Duration (Years)	№ of Districts	LLR (Log-Likelihood Ratio)	Mean Inside, Head/km^2^	Mean Outside, Head/km^2^
1	2015	2018	4	1	126.87	0.88	0.09
2	2010	2014	5	14	67.800	0.27	0.08
3	2010	2013	4	1	169.98	1.89	0.12
4	2010	2013	4	78	81.22	0.34	0.10
5	2010	2015	6	5	122.19	0.47	0.10
6	2016	2022	7	114	151.07	1.88	0.09
7	2010	2013	4	1	199.74	1.89	0.11
8	2010	2013	4	40	71.60	0.40	0.09
9	2010	2013	4	7	122.48	0.35	0.05
10	2010	2013	4	16	19.87	0.19	0.06
11	2016	2016	1	64	106.97	0.21	0.06
12	2011	2014	4	56	82.01	0.27	0.10
13	2010	2015	6	3	13.28	0.24	0.12
14	2010	2014	5	14	99.49	0.29	0.06
15	2010	2013	4	123	114.25	0.40	0.07
16	2010	2013	4	47	146.89	0.03	0.01
17	2019	2022	9	6	171.01	4.27	1.04
18	2015	2022	6	1	11.51	1.42	1.13
19	2020	2020	6	13	181.86	5.27	0.29
20	2016	2021	6	15	8.40	1.43	1.26

Note: LLR (log-likelihood ratio) is a parameter of a statistical test used to test limitations on the parameters of statistical models estimated based on sample rates.

## Data Availability

The data presented in this study are available upon request from the corresponding author.

## Supplementary Materials

The following supporting information can be downloaded at: https://www.mdpi.com/article/10.3390/ani13193081/s1, Table S1: Patterns of emerging hot spot analysis results.
