# Supplementary material for: Spatiotemporal Patterns of African Swine Fever in Wild Boar in the Russian Federation (2007–2022): Using Clustering Tools for Revealing High-Risk Areas
Source: Animals (Basel). 2023 Oct 2;13(19):3081. doi: 10.3390/ani13193081 (PMC10571777; doi:10.3390/ani13193081)
Supplement: Supplementary file 1 [file animals-13-03081-s001.zip › animals-2595751-supplementary.pdf]

Supplementary material

Table S1. Patterns of emerging hot spot analysis results.

Adopted from: ESRI. How Emerging Hot Spot Analysis works. Available online: https://pro.arcgis.com/en/pro-app/latest/tool-reference/space-time-pattern-mining/learnmoreemerging.htm)

Pattern	Pattern Name	Definition
	No Pattern Detected	Does not fall into any of the hot or cold spot patterns defined below.
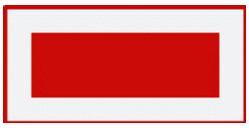	New Hot Spot	A location that is a statistically significant hot spot for the final time step and has never been a statistically significant hot spot before.
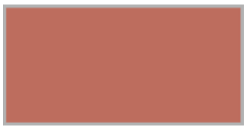	Consecutive Hot Spot	A location with a single uninterrupted run of at least two statistically significant hot spot bins in the final time-step intervals. The location has never been a statistically significant hot spot prior to the final hot spot run and less than 90 percent of all bins are statistically significant hot spots.
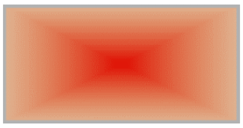	Intensifying Hot Spot	A location that has been a statistically significant hot spot for 90 percent of the time-step intervals, including the final time step.
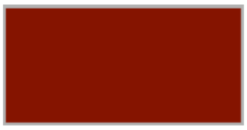	Persistent Hot Spot	A location that has been a statistically significant hot spot for 90 percent of the time-step intervals with no discernible trend in the intensity of clustering over time.
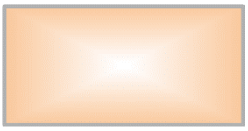	Diminishing Hot Spot	A location that has been a statistically significant hot spot for 90 percent of the time-step intervals, including the final time step. In addition, the intensity of clustering in each time step is decreasing overall and that decrease is statistically significant.
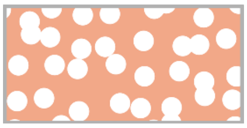	Sporadic Hot Spot	A statistically significant hot spot for the final time-step interval with a history of also being an on-again and off-again hot spot. Less than 90 percent of the time-step intervals have been statistically significant hot spots and none of the time-step intervals have been statistically significant cold spots.
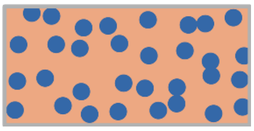	Oscillating Hot Spot	A statistically significant hot spot for the final time-step interval that has a history of also being a statistically significant cold spot during a prior time step. Less than 90 percent of the time-step intervals have been statistically significant hot spots.

	Historical Hot Spot	The most recent time period is not hot, but at least 90 percent of the time-step intervals have been statistically significant hot spots.
	New Cold Spot	A location that is a statistically significant cold spot for the final time step and has never been a statistically significant cold spot before.
	Consecutive Cold Spot	A location with a single uninterrupted run of at least two statistically significant cold spot bins in the final time-step intervals. The location has never been a statistically significant cold spot prior to the final cold spot run and less than 90 percent of all bins are statistically significant cold spots.
	Intensifying Hot Spot	A location that has been a statistically significant cold spot for 90 percent of the time-step intervals, including the final time step. In addition, the intensity of clustering of low counts in each time step is increasing overall and that increase is statistically significant.
	Persistent Cold Spot	A location that has been a statistically significant cold spot for 90 percent of the time-step intervals with no discernible trend in the intensity of clustering of counts over time.
	Diminishing Hot Spot	A location that has been a statistically significant cold spot for 90 percent of the time-step intervals, including the final time step. In addition, the intensity of clustering of low counts in each time step is decreasing overall and that decrease is statistically significant.
	Sporadic Hot Spot	A statistically significant cold spot for the final time-step interval with a history of also being an on-again and off-again cold spot. Less than 90 percent of the time-step intervals have been statistically significant cold spots and none of the time-step intervals have been statistically significant hot spots.
	Oscillating Hot Spot	A statistically significant cold spot for the final time-step interval that has a history of also being a statistically significant hot spot during a prior time step. Less than 90 percent of the time-step intervals have been statistically significant cold spots.
	Historical Hot Spot	The most recent time period is not cold, but at least 90 percent of the time-step intervals have been statistically significant cold spots.
